# Xiaoaiping Induces Developmental Toxicity in Zebrafish Embryos Through Activation of ER Stress, Apoptosis and the Wnt Pathway

**DOI:** 10.3389/fphar.2018.01250

**Published:** 2018-11-06

**Authors:** Juanjuan Li, Yun Zhang, Kechun Liu, Qiuxia He, Chen Sun, Jian Han, Liwen Han, Qingping Tian

**Affiliations:** ^1^Key Laboratory for Drug Screening Technology of Shandong Academy of Sciences, Biology Institute, Qilu University of Technology (Shandong Academy of Sciences), Jinan, China; ^2^School of Pharmaceutical Science, Shanxi Medical University, Taiyuan, China

**Keywords:** xiaoaiping, zebrafish embryos, developmental toxicity, swimming behavior, endoplasmic reticulum stress, apoptosis, Wnt pathway

## Abstract

The aim of the study was to determine the developmental toxicity of the traditional Chinese medicine Xiaoaiping (XAP) and to investigate its underlying mechanism of action. Zebrafish embryos were incubated with 0.4, 0.8, 1.2, and 1.6 mg/mL XAP. Endpoints such as mortality, hatching rate, malformation, body length, morphology score, swimming behavior, histological changes, reactive oxygen species (ROS) production, total superoxide dismutase (T-SOD) activity, and the mRNA expression of genes related to oxidative stress, endoplasmic reticulum (ER) stress, apoptosis, and the Wnt pathway were evaluated. Our results demonstrated that XAP exposure increased mortality and malformation and reduced the hatching rate. XAP resulted in severe malformation, including swim bladder deficiency, yolk retention, pericardial edema, and tail curvature. Histopathological analysis showed that XAP induced liver, heart and muscle injury. High doses (≥1.2 mg/mL) of XAP notably decreased the locomotor capacity of zebrafish. ROS generation was remarkably increased and T-SOD activity was decreased, confirming that oxidative stress was induced by XAP. The mRNA expression levels of ER stress-related genes (*chop, hspa5, hsp90b1, and perk*), apoptosis-related genes (*caspase-3*, *bax*, and *p53*) and *wnt11* were significantly upregulated by XAP exposure. The expression levels of the oxidative stress-related genes (*cat*, *sod1*, and *gstp2*), Wnt pathway-related genes (β-*catenin*, *wnt3a*, and *wnt8a*) and *bcl-2* initially increased and then decreased as the XAP exposure dose increased. In conclusion, we provide evidence for the first time that XAP can induce dose-related developmental toxicity, and ER stress, apoptosis and the Wnt pathway participate in the toxicity regulation.

## Introduction

XAP, which is an extract derived from *Marsdenia tenacissima*, has been widely used for the treatment of cancer in China. The dry stem of *M. tenacissima* (Roxb.) Wight et Arn. (Asclepiadaceae), commonly known as “Tong-Guan-Teng,” is an herb used in traditional Chinese medicine, and it was recorded in the 2010 edition of “Pharmacopeia of the People’s Republic of China” (Huang et al., 2013a). Tong-Guan-Teng has been known to have a broad spectrum of biological functions, such as heat-clearing and detoxification, relief of cough and asthma, as well as anti-inflammatory, anticancer and anti-HIV properties (Fan et al., 2015). Therefore, XAP, as an extract of *M. tenacissima*, also has a wide pharmacological action, particularly in gastric cancer, oesophageal cancer, lung cancer, and liver cancer (Han et al., 2012; Huang et al., 2013b; Pang et al., 2016). It has been applied alone or combined with chemotherapy or radiotherapy to treat cancers in China (Sun et al., 2010;Wang S. F. et al., 2011). In recent years, the study of XAP mainly concentrated on purification of the active ingredients to ascertain their pharmacological actions and mechanisms. However, its side effects are not well studied. It has been reported that XAP can result in diarrhea, measles, irritability, nausea, and myalgia (Che and Chen, 2015; Zhu and Liu, 2015). Its effects on pregnant patients are currently unknown; the effects of XAP on the growth of the fetus have not been investigated, and the mechanism of XAP-induced developmental toxicity remains unknown. Therefore, our study aims to investigate the developmental toxicity induced by XAP.

In the present study, we used the globally accepted zebrafish animal model. The zebrafish model is widely used in the fields of toxicology and biomedical research because it possesses an exceptional set of characteristics: (1) Due to its transparency, it is easy to observe the malformation of zebrafish embryos, which makes it an ideal model for drug toxicity research (He et al., 2014; Akhter et al., 2016); (2) Compared with traditional *in vivo* models such as mice, the zebrafish model has the advantages of low cost, a short life cycle, easy maintenance, high fecundity, smaller required amounts of test compound and high throughput (Wang Y. S. et al., 2011; Gao et al., 2016); (3) Toxicity assessment of zebrafish removes the effect of food due to the ease of husbandry and breeding within 7 days post fertilization (Spence et al., 2008); and (4) Unlike *Drosophila* and nematode species, zebrafish are vertebrates, and the genomic similarity between zebrafish and humans is at least 87% (Howe et al., 2013). The high level of gene homology results in the high conservation of signaling pathways between zebrafish and humans (Chakraborty et al., 2016). Zebrafish toxicity assessments of chemicals can predict potential toxicity in humans because the cardiovascular, nervous and digestive systems of this animal model are similar to those of humans (Hsu et al., 2007). Overall, the zebrafish is an excellent model for the study of developmental toxicology.

In the present study, the toxic effects of XAP on the development of zebrafish embryos were investigated. First, we examined the embryonic morphological defects caused by XAP. We then measured the expression levels of a series of genes associated with oxidative stress, ER stress, apoptosis and the Wnt pathway. By examining the results, this study attempts to assess the developmental toxicity of XAP and illustrate the mechanism of toxicity.

## Materials and Methods

### Reagents

XAP was purchased from Nanjing Sanhome Pharmaceutical Co., Ltd. (Batch No. 201601211; Nanjing, China). Based on high-performance liquid chromatographic (HPLC) testing, the contents of tenacissoside G, tenacissoside H and tenacissoside I in XAP were 10, 16, and 42 μg/mL, respectively. The chemical analysis of XAP constituents has been previously published (Li et al., 2015). Water soluble XAP extract was diluted in an aqueous solution identical in composition to the fish water (5 mM NaCl, 0.17 mM KCl, 0.4 mM CaCl_2_, 0.16 mM MgSO_4_) before the experiments. All other chemicals and reagents utilized in this study were of analytical grade.

### Zebrafish Maintenance and Embryo Collection

AB-strain zebrafish were kept under a 14/10 h light/dark cycle at constant temperature (28 ± 0.5°C) in a closed flow-through system with charcoal-filtered tap water. Brine shrimp were provided twice daily at 9:00 and 16:00. Adult male and female zebrafish in a 2:1 ratio were placed on opposite sides of a divider in a breeding tank the night before fertilization; the next morning, the zebrafish laid eggs by natural mating soon after the first light. Embryos were collected within 30 min after spawning and rinsed with fresh water three times. The clean embryos were moved to tanks with embryo medium and cultured at 28°C for the subsequent experiments. All experiments were carried out in compliance with the standard ethical guidelines and under control of Biology Institute, Qilu University of Technology of committee.

### Zebrafish Embryo Toxicity

At 6 hpf, the healthy embryos were randomly divided into 6-well plates (30/well) and were exposed to various concentrations of XAP (0, 0.4, 0.8, 1.2, 1.6, 2.4, and 3.2 mg/mL) in 5 mL of fish water, in triplicate. The exposure lasted for 120 h in an environment at 28 ± 0.5°C under the same light/dark cycle. Every 24 h the solutions were replaced, and dead embryos were discarded. The hatching rates and mortality were recorded, and the embryos were examined using a stereomicroscope (Olympus SZX16; Tokyo, Japan) at 24, 48, 72, 96, and 120 hpe.

### Morphology Score

Morphology scores were determined at 120 hpe. Nine endpoints, including body shape, somites, notochord, tail, fins, heart, face, brain, and pharyngeal arches/jaws, were examined to evaluate the phenotypes of the zebrafish, and eight larval specimens per group were used for scoring. Subsequently, the samples were anesthetized with 0.25 mg/mL tricaine and were observed and photographed using a stereomicroscope at a range of 1–10× magnification. The detailed General Morphological Scoring Criteria have been described in a previous work (Panzica-Kelly et al., 2010).

### Behavioral Analysis

At 120 h after being exposed to different concentrations of XAP, the larvae were subjected to behavioral tests. First, eight larvae per XAP concentration were rinsed twice in fresh water. They were then placed in 48-well plates with 0.5 mL of fish water and one larva per well and were incubated for 30 min at 28°C. Finally, we used Zebra Lab software (Viewpoint, France) to analyze the digital tracks over a span of 10 min.

### Heart Toxicity

Five dose levels, 0, 0.4, 0.8, 1.2, and 1.6 mg/mL, were selected for assessing the cardiovascular toxicity of XAP. After the zebrafish embryos were exposed to XAP from 6 to 120 hpf, 30 embryos from each group were randomly selected for visual observation and image acquisition of specific phenotypic endpoints under the dissecting stereomicroscope. The heart rate for each larva was recorded by counting the beats per minute under the dissecting stereomicroscope when the fish was stationary. The looping of the heart tube was quantified by measuring the distance between the sinus venosus (SV) and BA, as previously described with modifications. Image-Pro Plus software (Media Cybernetics, USA) was used to assess the length of a straight line between SV and BA.

### Histopathological Ultrastructural Evaluations

Histopathological evaluation was performed as previously described in detail (Menke et al., 2011). At 120 hpe, zebrafish exposed to XAP were fixed in 4% paraformaldehyde and were embedded in paraffin, sectioned and stained with haematoxylin and eosin (H&E). Additionally, electron microscopy examination was used to evaluate heart injury after zebrafish larvae were fixed in glutaraldehyde paraformaldehyde solution for 24 h.

### Measurement of ROS Generation and T-SOD Activity

DCF-DA (2′, 7′-dichlorodihydrofluorescein diacetate) (purchased from Beyotime Institute of Biotechnology) is a fluorescent probe dye that can detect the generation of reactive oxygen species (ROS) *in vivo*. We used a solution of 30 μM DCF-DA to treat larvae for 1 h in the dark at 28°C. The larvae were then washed with fish water, anesthetized with 0.16% tricaine and imaged using a fluorescence microscope (Olympus). Finally, we employed the ImageJ program to quantify the fluorescence intensity.

At 120 hpe, zebrafish exposed to XAP were washed three times using fish water. Cold saline was added to the zebrafish in a 1.5 ml tube at a ratio of 1:9 (g:mL) without any additional water. The samples were homogenized using automated tissue homogenization. The 10% homogenate was centrifuged at 2,500 rpm for 10 min at 4°C, and the supernatant was collected for testing. The total-superoxide dismutase (T-SOD) activity was detected using colourimetry according to the kit manufacturer’s instructions (Nanjing Jiancheng Bioengineering Institute, China).

### Total RNA Extraction and RT-PCR

A NanoMag Animal and Fish RNA Isolation Kit (Shannuo Scientific Company, Tianjin, China) was used to extract total RNA from 30 homogenized zebrafish larvae at 120 hpe. The synthesis of cDNA was carried out using PrimeScript RT Master Mix (Takara Biotechnology, Dalian, China) according to the manufacturer’s instructions. The detailed procedure for RT-PCR was drawn from a previous work (Zhang et al., 2016). Each gene in the present study was assessed in triplicate. The sequences of primers for the real-time PCR are shown in Table 1.

**Table 1:** Quantitative PCR primer sequences.

Gene	Primer orientation	Neucleotide sequence
β-*actin*	forward	5′-AGAGCTATGAGCTGCCTGACG-3′
	reverse	5′-CCGCAAGATTCCATACCCA-3′
*sod1*	forward	5′-GGCCAACCGATAGTGTTAGA-3′
	reverse	5′-CCAGCGTTGCCAGTTTTTAG-3′
*cat*	forward	5′-AGGGCAACTGGGATCTTACA-3′
	reverse	5′-TTTATGGGACCAGACCTTGG-3′
*gstp2*	forward	5′-CACAGACCTCGCTTTTCACAC-3′
	reverse	5′-GAGAGAAGCCTCACAGTCGT-3′
*xbp1s*	forward	5′-CAAAGGAGCAGGTTCAGGTAC-3′
	reverse	5′-GGAGATCAGACTCAGAGTCTG-3′
*perk*	forward	5′-TGGGCTCTGAAGAGTTCGAT-3′
	reverse	5′-TGTGAGCCTTCTCCGTCTTT-3′
*hspa5*	forward	5′-CAGATCTGGCCAAAATGCGG-3′
	reverse	5′-GGAACAAGTCCATGTTGAGC-3′
*chop*	forward	5′-CACAGACCCTGAATCAGAAG-3′
	reverse	5′-CCACGTGTCTTTTATCTCCC-3′
*caspase-3*	forward	5′-CCGCTGCCCATCACTA-3′
	reverse	5′-ATCCTTTCACGACCATCT-3′
*bax*	forward	5′-GGCTATTTCAACCAGGGTTCC-3′
	reverse	5′-TGCGAATCACCAATGCTGT-3′
*bcl-1*	forward	5′-TCACTCGTTCAGACCCTCAT-3′
	reverse	5′-ACGCTTTCCACGCACAT-3′
*p53*	forward	5′-ACCACTGGGACCAAACGTAG-3′
	reverse	5′-CAGAGTCGCTTCTTCCTTCG-3′
β-*catenin*	forward	5′-CATTACAACTCTCCACAACC-3′
	reverse	5′-CAGATAGCACCTTCAGCAC-3′
*wnt3a*	forward	5′-CACCTCCCATCCCTTCCTA-3′
	reverse	5′-CCGTTCTGCTCAAGTGTCCT-3′
*wnt8a*	forward	5′-TTGTCCGCAACTAAAGTGGT-3′
	reverse	5′-CCTGGTAACGGTTTGAGT-3′
*wnt11*	forward	5′-TTGTCCGCAACTAAAGTGGT-3′
	reverse	5′-TCATTTGCAGACGTATTTC-3′

### Statistical Analyses

The data analyses were conducted using Statistical Package for the Social Sciences (SPSS) and Excel software. The data are expressed as the means ± standard error (SE). Significant differences between the mean values were analyzed using one-way analysis of variance (ANOVA-SNK). The differences were considered to be statistically significant if the *P*-values were less than 0.05(^∗^) or 0.01(^∗∗^).

## Results

### Mortality

**FIGURE 1:**
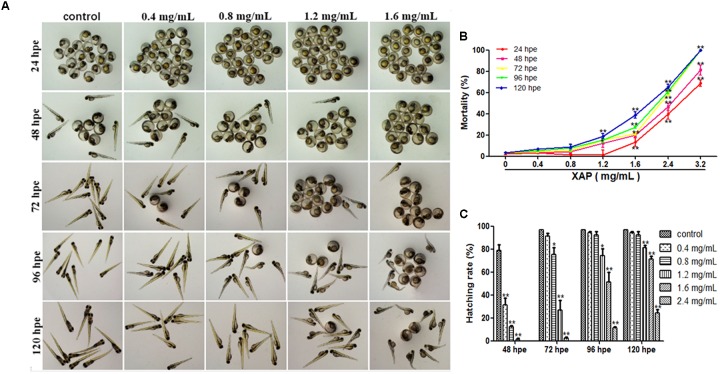
Phenotypes, mortality, and hatching rate of embryos following aqueous exposure to XAP from 24 to 120 hpf. **(A)** The phenotypes of embryos in the unexposed group and XAP exposure groups. **(B)** The mortality rate in zebrafish embryos exposed to XAP. **(C)** The hatching rate in zebrafish embryos exposed to XAP. ^∗^*P* < 0.05, ^∗∗^*P* < 0.01 versus control.

The cumulative mortality of the embryos after XAP exposure is shown in Figure 1B. Mortality was recorded at 24, 48, 72, 96, and 120 hpe in order of increasing concentration. The median lethal concentration (LC_50_) of XAP was calculated by sigmoidal regression using SPSS software based on the zebrafish lethality curves (Figure 1B). The LC_50_ was 2.66 mg/mL at 24 hpe, 2.31 mg/mL at 48 hpe, 1.92 mg/mL at 72 hpe, 1.91 mg/mL at 96 hpe, and 1.79 mg/mL at 120 hpe.

As Figure 1B shows, significant increases in mortality were observed in embryos at concentrations higher than 1.6 mg/mL at 24 and 48 hpe, and all fish died with exposure concentrations of 3.2 mg/mL at 72 hpe. At 120 hpe, 2.4 mg/mL (65.56 ± 2.22%, *p* < 0.01), and 1.6 mg/mL (28.89 ± 2.94%, *p* < 0.01) had significantly increased mortality compared with the control group (3.33 ± 0.00%). Meanwhile, doses lower than 0.8 mg/mL had no significant impact on mortality from 24 to 120 hpe.

### Hatching Rate

Hatching is known to be a critical period in zebrafish embryogenesis; therefore, the hatching rate is one of the most important indices with which to evaluate the developmental toxicity of XAP in zebrafish. Generally, embryos started to hatch by 48 hpe and finished by 96 hpe. Our results showed that 79 and 97% of the control zebrafish embryos had hatched by 48 hpe and 72 hpe, respectively. However, the embryo-hatching rate was significantly reduced in the XAP exposure groups, with hatching rates of 51.11 ± 8.68% and 11.11 ± 1.11% in the 1.6 and 2.4 mg/mL XAP-treated groups by 96 hpe, respectively. Only 25% of the embryos hatched naturally by 120 hpe in the group incubated with 2.4 mg/mL of XAP (Figure 1C). These data indicated a remarkable dose- and time-dependent decrease in the hatching rate in the XAP-treated groups compared with those of the control.

### Malformations

Figure 1A shows the development of the zebrafish embryos exposed to XAP from 2 to 120 hpe. Many morphogenetic abnormalities, including spinal curvature, edema and eye defects, were observed in the 1.6 mg/mL XAP-treated group at 120 hpe (Figure 1A). Additionally, we found that the XAP-treated (0.8, 1.2, or 1.6 mg/mL) groups showed an apparent delay in hatching compared with the control group from 48 to 120 hpe.

### Malformation Scores and Rates

**FIGURE 2:**
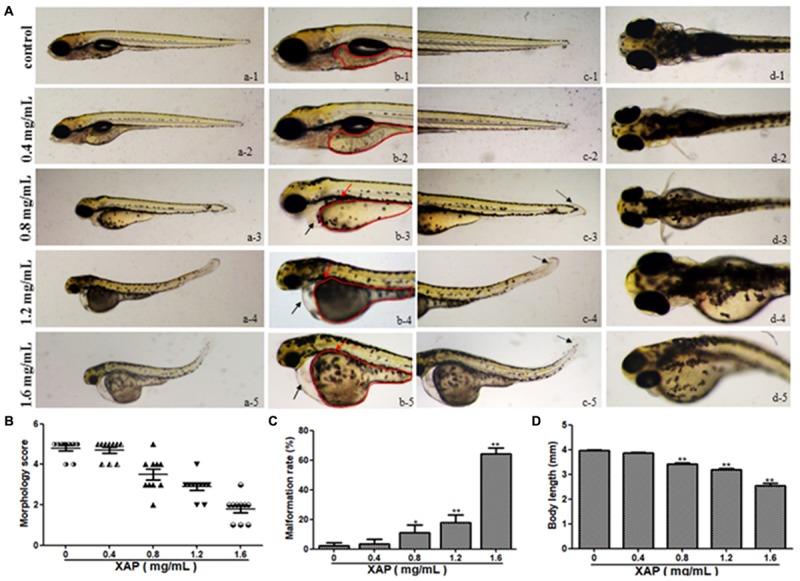
Body morphology effect of XAP exposure in zebrafish embryos at 120 hpe. **(A)** Representative lateral and ventral views of zebrafish larvae. Swim bladder deficiency is indicated by a solid red arrow. Yolk retention is indicated by the red outline. Pericardial edema is indicated by a solid black arrow. A slightly curved tail is indicated by a dotted black arrow. **(B)** Morphological scoring of XAP-exposed zebrafish embryos at 120 hpe. **(C)** Malformation rate of XAP-exposed zebrafish embryos at 120 hpe. **(D)** Body length of XAP-exposed zebrafish embryos at 120 hpe. ^∗^*P* < 0.05, ^∗∗^*P* < 0.01 versus control.

We tested phenotypic defects caused by XAP at 120 hpe. Compared with the control group, the malformation rate of the 0.4 mg/mL XAP group showed no significant change. In the 0.8 mg/mL XAP group, slight malformations were observed, mainly consisting of swim bladder deficiency, subtle yolk retention, and pericardial edema. When the XAP concentration reached 1.2 mg/mL, developmental abnormalities became more apparent, including spinal curvature, swim bladder absence, yolk retention, pericardial edema, and tail malformation. In the 1.6 mg/mL XAP group, multiple malformations were found simultaneously in individual fish, such as severe pericardial edema, tail curvature, and missing somites. As shown in Figure 2A, the vehicle control zebrafish exhibited clear and regular somites, while ill-defined somites were observed in the XAP exposure groups (1.2 and 1.6 mg/mL). As is shown in Figure 2B, it is clear that the morphology score decreases as the XAP exposure concentration increases.

The malformation rates of XAP-treated larvae at 120 hpe are shown in Figure 2C. There were significant increases in the rates of malformation in the XAP-treated groups compared with the control group, with malformation rates of 11.11 ± 5.09%, 17.78 ± 5.09%, and 64.44 ± 3.85% in the 0.8, 1.2, and 1.6 mg/mL XAP-treated groups, respectively (Figure 2C).

### Body Length

The body lengths of the larvae were measured at 120 hpe to assess the degree of development (Figure 2D). The body lengths of the larvae at 120 hpe were significantly reduced in a dose-dependent manner, especially with 0.8 mg/mL (3.37 ± 0.02, *p* < 0.01), 1.2 mg/mL (3.18 ± 0.06, *p* < 0.01) and 1.6 mg/mL (2.53 ± 0.11, *p* < 0.01) treatment, compared with the control group (4.00 ± 0.02), which indicated that XAP significantly inhibited larval growth.

### Swimming Behavior

**FIGURE 3:**
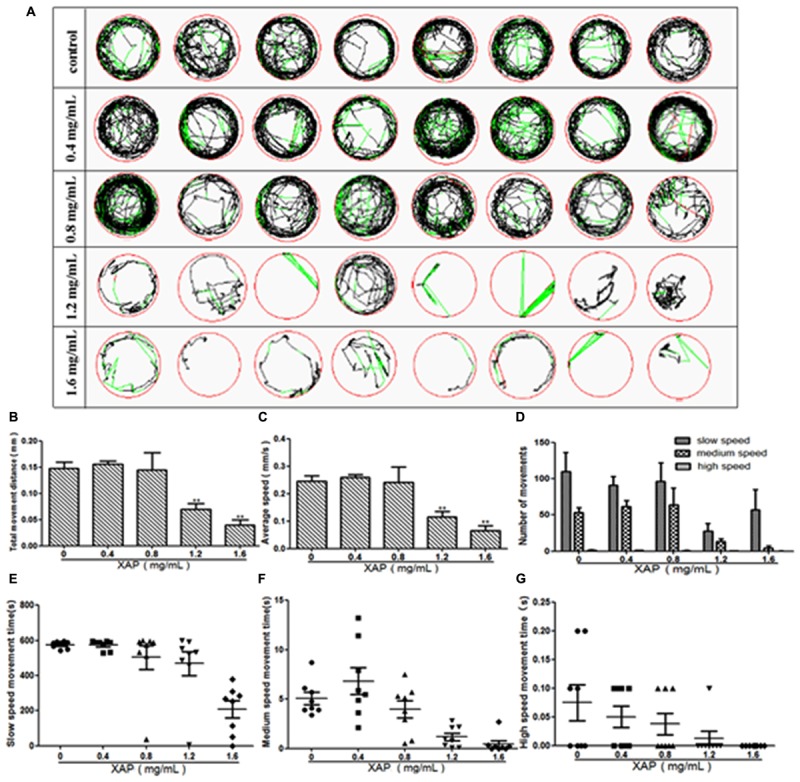
Reduction of locomotor capacity following exposure to XAP. **(A)** Digital tracks of larvae from the 0, 0.4, 0.8, 1.2, and 1.6 mg/mL exposed groups at 120 hpe. **(B)** Total movement distance, **(C)** average speed, **(D)** number of movements, and **(E–G)** the average speed of larvae exposed to 0, 0.4, 0.8, 1.2, and 1.6 mg/mL XAP at 120 hpe. ^∗^*P* < 0.05, ^∗∗^*P* < 0.01 versus control. s, second.

We performed behavioral tests on the larvae at 120 hpe. The digital tracks are shown in Figure 3A. On the digital track map, the red lines, green lines and black lines indicate high-speed movement, medium-speed movement and slow-speed movement, respectively. As is shown in Figure 3D, there is no significant shanges for the number of movements of zebrafish in slow speed, medium speed and high speed. We found that most of the movement time of the larvae was spent at medium and slow speed in both the exposed and unexposed groups; however, the high-speed movement time, the medium-speed movement time and the slow-speed movement time were all significantly reduced in both the 1.2 and 1.6 mg/mL exposed groups compared with the unexposed group in Figures 3E–G. Additionally, the larvae from the groups exposed to 0.8, 1.2, or 1.6 mg/mL XAP showed notable decreases in average speed in Figure 3C. The total distance was significantly reduced at concentrations of 1.2 mg/mL (69.53 ± 11.63, *p* < 0.01) and 1.6 mg/mL (39.39 ± 10.38, *p* < 0.01) compared with the control group (147.76 ± 11.41) in Figure 3B. These results demonstrated that XAP lessened the locomotor capacity of the zebrafish larvae.

### Histological Analysis

**FIGURE 4:**
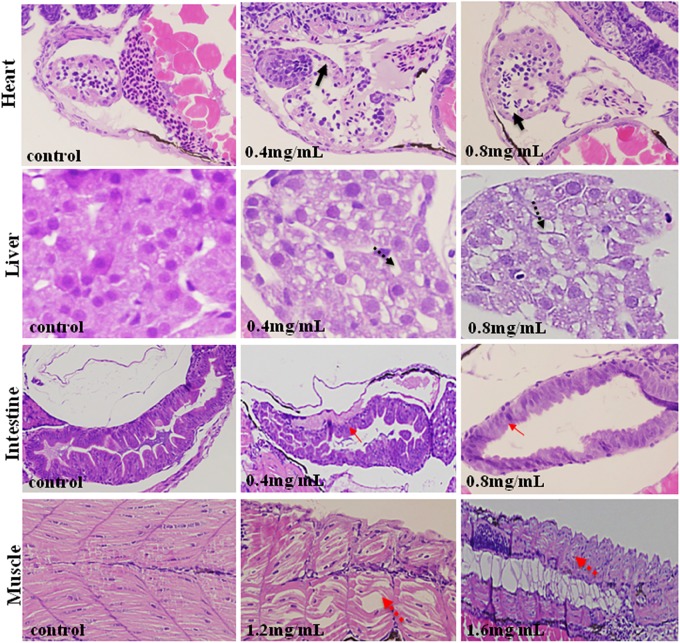
Histopathological changes in the hearts, livers, intestines and muscles of zebrafish larvae exposed to XAP at 120 hpe. A thinner heart wall is indicated by a solid black arrow. Loose hepatocytes are indicated by a dotted black arrow. Frayed gut villi is indicated by a solid red arrow. Muscle atrophy is indicated by a dotted red arrow.

As is shown in Figure 4, XAP had obvious effects on the heart, liver, intestines and muscle, including thinner heart walls, pericardial edema, loose hepatocytes, frayed gut villi and muscle atrophy.

### Heart Toxicity Assessment

**FIGURE 5:**
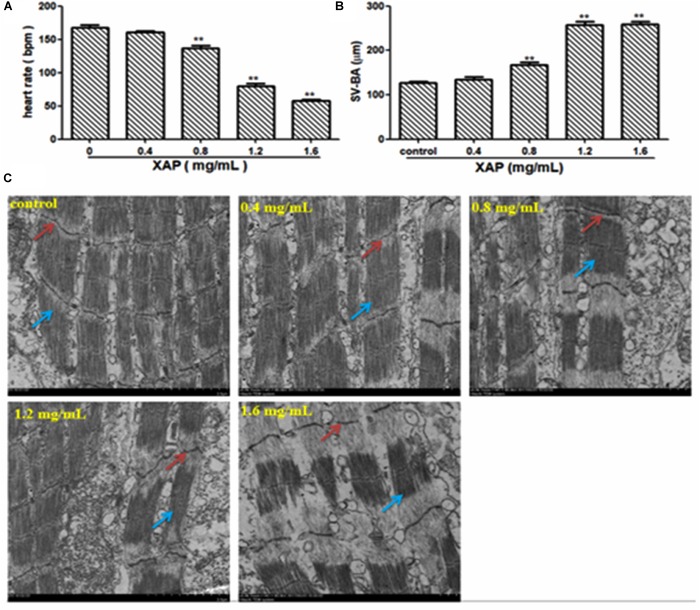
Heart toxicity effects of XAP in zebrafish embryos at 120 hpe. **(A)** The heart rate (beats/minute) in zebrafish larvae. **(B)** The SV-BA distance (μm) in zebrafish larvae. **(C)** The TEM image of zebrafish heart. Striated muscle is indicated by a solid red arrow, cardiac muscle fiber is indicated by a solid blue arrow. ^∗^*P* < 0.05, ^∗∗^*P* < 0.01 versus control. SV, sinus venous; BA, bulbus arteriosus; TEM, transmission electron microscope.

As shown in Figure 5A, heart rate changes were found in a dose-dependent manner with XAP exposure, and a statistically significant decrease occurred at XAP doses of 0.8 mg/mL and above compared with the control group (*p* < 0.01). The distance between the SV and BA provides a marker of the looping of the heart tube into two distinctive heart chambers. Our study demonstrated that XAP exposure caused a significant increase in the SV-BA distance in a dose-dependent manner (Figure 5B). Figure 5C shows that the structure of the zebrafish heart was damaged. Zebrafish treated with XAP (0.8, 1.2, and 1.6 mg/mL) showed significant structural abnormalities in the heart, such as myofibrillar fragmentation and striated muscle disorder. These results suggested that XAP might induce heart toxicity in zebrafish.

### ROS and T-SOD Measurement

**FIGURE 6:**
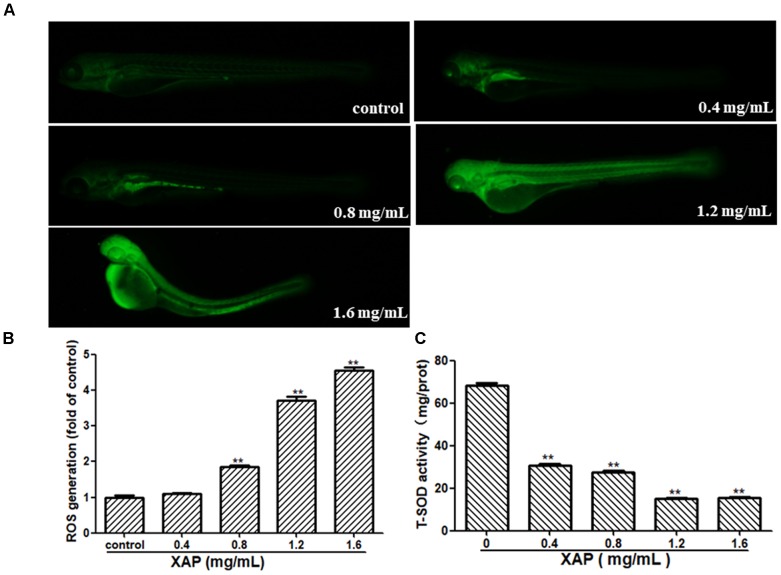
Changes in ROS levels induced by XAP at 120 hpe. **(A)** ROS generation was identified as green fluorescence on a black background after XAP exposure. **(B)** The quantitative analysis of ROS generation after XAP exposure. **(C)** The change of T-SOD activity after XAP exposure. ^∗^*P* < 0.05, ^∗∗^*P* < 0.01 versus control.

As shown in Figure 6A, we observed that there was no fluorescence in the control group, whereas there was a significant dose-dependent increase in fluorescence in the XAP-treated groups. This was consistent with the result in Figure 6B, showing that the generation of ROS followed an ascending trend in the treated groups (0.8, 1.2, and 1.6 mg/mL); meanwhile, the T-SOD activity was significantly decreased, which suggests that XAP likely induces oxidative stress in zebrafish in Figure 6C.

### Gene Expression

**FIGURE 7:**
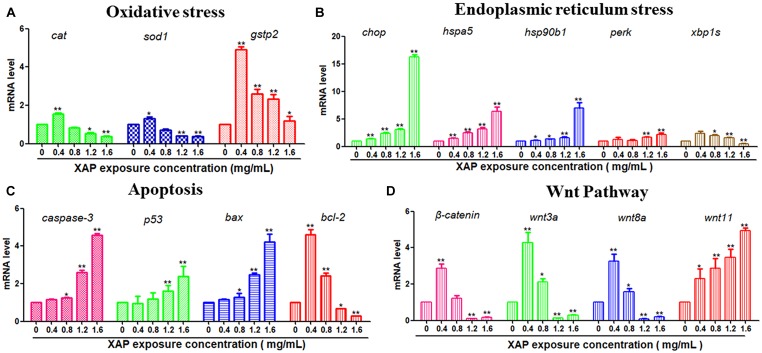
Related gene expression levels of **(A)** Oxidative stress, **(B)** Endoplasmic reticulum stress, **(C)** Apoptosis, and **(D)** Wnt pathway in zebrafish embryos exposed to XAP at concentrations of 0, 0.4, 0.8, 1.2, and 1.6 mg/mL at 120 hpe. The expression levels of mRNA are represented as the fold change from the control group. ^∗^*P* < 0.05, ^∗∗^*P* < 0.01 versus control.

To investigate the possible mechanisms of the toxic effects induced by XAP, we used RT-PCR to examine the mRNA expression levels of larvae exposed by different XAP concentrations from 6 to 120 hpf. The RT-PCR results showed that the expression levels of the oxidative stress-related genes (*cat*, *sod1*, and *gstp2*) were upregulated in the XAP exposure group (0.4 mg/mL) compared with the control group, while they were downregulated in the other groups (1.2 and 1.6 mg/mL). The mRNA expression levels of ER stress-related genes (*chop, hspa5, hsp90b1*, and *perk*) and apoptosis-related genes (*caspase-3*, *p53*, and *bax*) increased with the increased XAP exposure dose. The mRNA expression level of *bcl-2* was downregulated with increasing XAP exposure doses. The mRNA expression levels of Wnt pathway-related genes (β-*catenin*, *wnt3a*, and *wnt8a*) first increased and then decreased as the XAP exposure dose increased; however, the expression of *wnt11* increased significantly with the increase of XAP exposure concentration in Figure 7.

## Discussion

In the present study, we revealed that XAP resulted in developmental toxicity in wild-type AB-strain zebrafish embryos, particularly morphological abnormalities, and delayed hatching. The 0.4, 0.8, 1.2, 1.6, and 2.4 mg/mL doses chosen for the toxicity studies in zebrafish model ranged from no effect on the development to clear the toxic effect on development. XAP treatment at 0.4 mg/mL did not produce noticeable signs of toxicity, whereas doses of 1.2 and 1.6 mg/mL obviously reduced hatching rate at 72 hpe and significantly decreased body length at 120 hpe. This was the first time that we had employed zebrafish to investigate the developmental toxicity of XAP. Hatching is a critical period of zebrafish embryogenesis; therefore, the reduced hatching rate was attributable to the structural and functional disturbances that occurred during embryonic development (Samaee et al., 2015; Liu et al., 2016). In addition, the inhibition of mitosis or the suppression of embryogenesis (Ismail and Yusof, 2011) or the inability of the emerging larvae to break the eggshell (Smha and Kanamadi, 2000) also likely contributed to the developmental delay. A significant dose-dependent decrease in hatching rate was found in our study. This hatching delay is also an important indicator of developmental toxicity. In the study, XAP treatment induced embryonic teratogenesis, including pericardial edema, spinal curvature, uninflated swim bladders and bent tails. Spinal teratogenesis, the most common type of malformation, might be associated with an imbalance of ions (such as calcium and phosphorus) or a decrease in myosin, both of which are essential for development (Oliveira et al., 2009). In addition, the reduction of body length at 120 hpe indicated that XAP exposure could inhibit the growth of zebrafish larvae, which was most likely explained by delayed deciduation. Taken together, these results demonstrated that developmental toxicity of zebrafish was induced by XAP exposure.

The behavioral analysis of zebrafish locomotion is very intuitive, simple, and quick (Lieschke and Currie, 2007). As yet, the behavioral aspects of XAP exposure have not been reported. In this study, the swimming behavior was evaluated by determining the total movement distance, velocity, total amount of turning, high-speed movement time, medium-speed movement time, and slow-speed movement time (Sun et al., 2016). The total movement distance, velocity, and total amount of turning decreased significantly in the exposed larvae in a dose-dependent manner. Combined with the digital track maps, these results showed that larvae of the two exposed groups (1.2 and 1.6 mg/mL) were less active compared with the control group. Three swimming speeds, namely, slow (<2 mm/s), medium (2–5 mm/s), and high (>5 mm/s), were used to describe the movement trajectory of the larval zebrafish (Winter et al., 2008). Most of the movement time of the larvae was concentrated at the medium and slow speeds in the exposed groups, which indicated that most larvae preferred to swim at a medium to low speed after XAP exposure. This is the first study to demonstrate that XAP exposure at high doses results in reduced locomotion ability. It has been reported that alterations in larval behavior might be related to central nervous system (CNS) damage (Duan et al., 2013; Sun et al., 2016). Additionally, the deficiency of the swim bladder may be the main cause of the decrease in movement ability. Further investigations will be required to provide evidence regarding this hypothesis.

It has been reported that there are two main mechanisms of drug-induced oxidative stress – an increase in ROS production and a reduction of cellular antioxidant defenses (Baillie and Rettie, 2011; Leung et al., 2012). ROS is the key factor promoting oxidative stress. The excessive production of ROS *in vivo* will induce oxidative stress (Wijesinghe et al., 2013) because oxygen free radicals can be excessively combined with SOD, CAT and GSH, which will make the body’s antioxidant protection mechanism imbalanced. SOD is a key enzyme in the antioxidant system and can inhibit externally induced oxidative damage to the body, remove ROS and prevent lipid peroxidation (Jin et al., 2015). *gstp2* is a member of the GST Pi family, and it plays a vital antioxidant role in the antioxidant system by removing ROS with glutathione (Dong et al., 2013). In our study, the content of ROS in the zebrafish was increased. The RT-PCR results showed that the expression levels of *sod1*, *cat*, and *gstp2* in the 0.4 mg/mL group increased, and the expression levels of *sod1*, *cat* and *gstp2* in the 1.2 and 1.6 mg/mL groups decreased. The possible reason for this is that negative feedback regulation plays a vital role in low concentration stimulation, and the ability of antioxidant defenses was attenuated in the high-exposure groups. These results indicated that oxidative stress was activated in zebrafish embryos exposed to XAP and played an important role in the developmental toxicity of XAP.

There is a close relationship between oxidative stress and ER stress. ER stress can increase the content of ROS and induce oxidative stress (Landau et al., 2013), while oxidative stress can hinder the proper folding and transport of proteins, calcium homeostasis and stimulate ER stress (Malhotra and Kaufman, 2007a). Therefore, we detected the expression of ER stress-related genes. *chop* is a key mediator of ER stress and is induced when ER stress is prolonged or too severe for the organism to resolve (Malhotra and Kaufman, 2007b). *hspa5* is a zebrafish homologue of the molecular chaperone *grp78*, and *hsp90b1* is a zebrafish homologue of the molecular chaperone *grp94* (Luan et al., 2009; Walter and Ron, 2011). *grp78* is a molecular chaperone and acts as a central regulator for ER homeostasis and anti-apoptotic mechanisms (Pfaffenbach and Lee, 2011; Zhu et al., 2013). Under normal circumstances, *grp78* binds to *perk*, *atf6*, and *ire1* and keeps them inactive during unstressed conditions (Wang and Kaufman, 2012). However, the *grp78*-kinase complexes are dissociated and release their kinases so that they can activate unfolded protein reaction (UPR) signaling when unfolded or misfolded proteins accumulate in the ER (Luan et al., 2009). Previous reports have demonstrated that the *hsp90b1* gene plays a vital role in protein folding (Komoike and Matsuoka, 2013). *perk* is an ER-associated transmembrane serine/threonine protein kinase whose activation can induce transcription of other UPR-dependent genes (Harding et al., 1999). In our study, we found that the expression levels of *chop*, *hspa5, hsp90b1*, and *perk* were significantly upregulated, which indicated that ER stress was activated in zebrafish embryos against XAP-induced toxicity.

*chop* is an endoplasmic reticulum stress-induced apoptosis marker protein. When ER stress is activated, *chop* is significantly increased. The increased chop will cause an increase in *bax*, activation of *caspase-3* and *bax*, and induce apoptosis in related cells (Oyadomari and Mori, 2004). *p53* is a multifunctional regulation protein. Its activation can regulate the expression of apoptosis-inducing target genes (Amaral et al., 2010), and *bcl-2* is a type of apoptosis inhibition factor (Siddiqui et al., 2015). In this experiment, the exposure of XAP can significantly increase the expression levels of *p53, bax*, and *caspase-3*. Additionally, the *bcl-2* expression level of the 0.8 mg/mL group was raised, while the expression level of the high concentration (1.2 and 1.6 mg/mL) exposure groups was lowered. These results indicated that apoptosis was induced by XAP in zebrafish.

The Wnt signaling pathway, including the classical and non-classical pathways, plays an important role in the early stages of vertebrate embryo development. β-*catenin*, a key regulator of this classical signaling pathway, is associated with developmental malformations (Chinison et al., 2016). *wnt3a* and *wnt8a* are involved in the regulation of the classical Wnt pathway, and inhibition of *wnt3a* can cause deformities of the zebrafish body joints and the posterior structure of the body. Inhibition of *wnt8a* results in the absence of tissue near the anterior plate and axis of the spinal cord (Shimizu et al., 2005; Wen et al., 2016). Our results showed that the expression levels of *wnt3a* and *wnt8a* were significantly downregulated in the 1.2 and 1.6 mg/mL XAP groups, which was consistent with the phenotype of developmental toxicity (Figure 2A). *wnt11*, which regulates late-stage heart development and participates in the regulation of cardiac circulation and differentiation, is a key regulatory factor in the non-classical pathway (Pandur et al., 2002). In general, the expression of *wnt11* is high in the developmental stages before the maturity of heart development. Our research found that the expression of *wnt11* significantly and gradually increased with the increase of XAP exposure concentration, indicating that the development of zebrafish heart was delayed in the XAP exposure groups.

In conclusion, XAP caused a dose-dependent increase in developmental damage in zebrafish embryos, indicated by an increase in mortality and malformations, a decreased body length, and a delayed hatching period. Meanwhile, XAP induced oxidative stress, which affected ROS generation and T-SOD activity. Moreover, changes in the transcription levels of several representative genes related to oxidative stress, ER stress, apoptosis and the Wnt pathway suggested that XAP was able to induce developmental toxicity in zebrafish embryos through activation of oxidative stress and ER stress, further inducing apoptosis. Additionally, the developmental toxicity induced by XAP was also associated with the Wnt signaling pathway. The findings from this study will help to elucidate the mechanisms of XAP-induced developmental damage.

## Author Contributions

JL, CS, JH, and YZ conducted the experiments. JL and YZ drafted the manuscript. KL, and QH conceived the experiments. YZ, QT, and LH provided funding sources and edited the paper. All authors read and approved the submitted version.

## Conflict of Interest Statement

The authors declare that the research was conducted in the absence of any commercial or financial relationships that could be construed as a potential conflict of interest. The reviewer FW and handling Editor declared their shared affiliation.

## Abbreviations

BA: bulbus arteriosus
ER: endoplasmic reticulum
hpe: hours post exposure
hpf: hours post fertilization
SV: sinus venosus
XAP: xiaoaiping.
